# Glomus tumors around or in the knee: a case report and literature review

**DOI:** 10.1186/s12893-022-01545-8

**Published:** 2022-03-16

**Authors:** Yingjie Wang, Tian Li, Zehui Lv, Yanyan Bian, Bin Feng, Yong Liu, Xi Zhou, Xisheng Weng

**Affiliations:** 1 Department of Orthopedic Surgery, State Key Laboratory of Complex Severe and Rare Diseases, Peking Union Medical College Hospital, Peking Union Medical College & Chinese Academy of Medical Sciences, No. 1 Shuaifuyuan Rd, Beijing, 100730 China; 2 School of Basic Medicine, Fourth Military Medical University, No. 169 Changle West Rd, Xi’an, 710032 China

**Keywords:** Glomus tumor, Knee, Diagnosis, Treatment, Prognosis

## Abstract

**Background:**

Glomus tumors commonly affect the extremities, especially subungual. And glomus tumors rarely occur around knee, which are often misdiagnosed. A lack of experience with glomus tumors is likely the cause.

**Case presentation:**

A 42-year-old female presented with continuous dull pain of right knee for the past 7 years. Severe pain was experienced after walking a few hundred meters or climbing up or down stairs. The patient had a slight limp, and the lateral superior aspect of her right knee was tender to palpation. The range of motion and skin around her right knee were normal. Magnetic resonance imaging revealed a well-defined abnormal lesion confluent with the periosteum on the femoral lateral supracondylar. She was finally diagnosed with glomus tumor according to pathological results. After surgery, the pain disappeared, and the patient was discharged three days postoperatively. At the 18-month follow-up visit, the patient reported sustained pain relief, and regular follow-ups were continued. Additionally, 30 published reports documenting 36 cases of glomus tumors around the knee were reviewed, which showed that 20% of all reported cases of glomus tumor around the knee had a history of trauma. The median age for male with glomus tumor was greater than that of female; however, the median duration of illness between the two groups was equivalent. The mean diameters of glomus tumors ranged from 4 to 65 mm, and locations around the knee included the knee joint cavity, soft tissue (e.g. popliteal fossa, patellar tendon, iliotibial band, and Hoffa’s fat pad), distal femur, and proximal tibia.

**Conclusion:**

Literature review demonstrated that no significant differences were found between male and female with glomus tumor in regard to location (left or right side) and illness duration. It was noting that a history of trauma may be a cause of glomus tumor and approximate 94.4% of glomus tumors was benign. The most effective therapy accepted for glomus tumors is complete surgical excision, and recurrence was rare after complete surgical excision.

## Background

Glomus tumors (GT), derived from the glomus body, are rare, benign tumors composed of cells similar to modified smooth muscle cells in normal hemangiospheres [1]. GTs are usually located in skin areas that are rich in blood vessels, such as the subungual area, or the deep dermis of the palms, wrists, forearms, and feet. Finger and toe GTs are seen in about 5% of patients with neurofibromatosis type 1 (NF1), which are considered NF1-related tumors [2–4]. Currently, the clinical diagnosis of GT is based on the classic triad of pain and tenderness to palpation and hypersensitivity to cold. Definitive diagnosis requires resection of the tumor for histopathological examination. Complete surgical excision is widely used to treat GT. Although most GTs are commonly considered benign, several malignant [5, 6], and uncertain potentially malignant GTs have been reported [7–9].

GTs are easily missed by orthopedic surgeons, or misdiagnosed as other diseases including osteoarthritis, soft tissue injuries, popliteal fossa cyst, neurogenic tumors, vascular tumors, pigmented nevi, epidermal cysts, lipomas, leiomyomas, and sarcoidosis [10]. Misdiagnosis may result from a lack of information regarding GTs in uncommon sites. To our knowledge, only 36 cases of around the knee GT have been reported, most likely due to low incidence and high rate of missed tumors or incorrect diagnoses.

Herein, we present a case report of GT around the knee. In order to provide more information concerning GTs in uncommon sites without typical symptoms, we review the current literature, highlighting etiology, clinical manifestations, affected areas, imaging results, age differences, affected sides, and duration of illness in males and females. Finally, we investigate the management and prognosis for GTs.

## Case presentation

A 42-year-old female presented with continuous dull pain of the right knee for the past 7 years. She denied a history of trauma, and spontaneous severe pain or cold sensitivity had not been observed. However, severe pain was experienced after walking a few hundred meters, or climbing up or down stairs. The pain was relieved after rest, but occasionally worsened at night. She had been initially diagnosed with osteoarthritis 7 years previously, and at that time sodium hyaluronate was proposed, but refused by the patient. Soon thereafter, the patient was diagnosed at another medical center with synovitis of the knee. Although diclofenac sodium was administrated, her symptoms were not significantly relieved. In addition, 3 years prior, she had not received a confirmed diagnosis at two other hospitals.

**Fig. 1:**
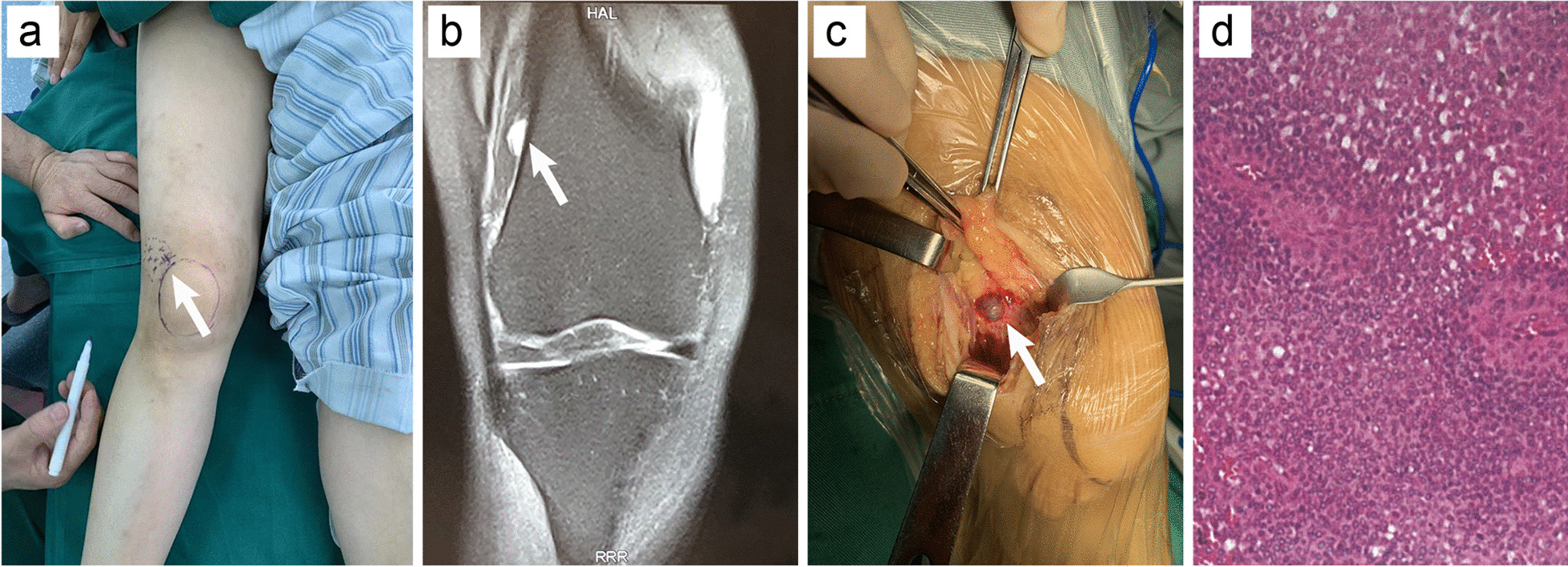
**a** The area of tenderness above the lateral side of the right tibia. **b** A well-defined abnormal lesion on the femoral lateral supracondylar confluent with the periosteum of the femur. **c** A purple mass located on the anterolateral aspect of the femur periosteum. **d** H&E staining, original magnification × 100.

The patient had a slight limp, and upon physical examination, the lateral superior aspect of her right knee was tender to palpation, initially preventing thorough evaluation of the area. Subsequently, an area of unbearable tenderness, with a diameter of ~ 50 mm, was observed ~ 25 mm above the lateral side of the right tibia (Fig. 1a). The range of motion and skin around her right knee were normal. A neurovascular examination was unremarkable, and radiographs failed to demonstrate any abnormality. However, magnetic resonance imaging (MRI) of the right knee revealed a well-defined abnormal lesion on the femoral lateral supracondylar, confluent with the periosteum of the femur (Fig. 1b). Examination of T2-weighted images revealed that the lesion was homogeneously hyperintense. Moreover, a neoplasm was discovered on the femoral medial supracondylar, implying osteochondroma.

Surgical exploration was suggested by our team and accepted by the patient. During surgery, a purple mass was located on the anterolateral aspect of the femur periosteum and did not appear to scallop the periosteum or outer cortex (Fig. 1c). The mass was totally excised and measured 10 × 7 × 4 mm. Hematoxylin–eosin (H&E) staining revealed numerous mononucleated globus cells, with pale and eosinophilic cytoplasm, as well as thin- and thick-walled vessels. Moreover, the results of immunohistochemical staining with monoclonal antibodies was positive for vimentin, h-caldesmon, and smooth muscle actin, but negative for cytokeratin, S-100, CD34, CD31, and desmin. The Ki-67 proliferation index was 10%. Pathological results confirmed that the mass was a GT (Fig. 1d). After surgery, the pain disappeared, and the patient was discharged three days postoperatively. At the 18-month follow-up visit, the patient reported sustained pain relief, and regular follow-ups were continued.

## Discussion and conclusions

### Literature search and selection

PubMed, Embase, and Web of Science databases were used for literature search. The search terms were (“Bones and Bone Tissue” OR “Bones and Bone” OR “Bone Tissue” OR “Bone Tissues” OR “Tissue, Bone” OR “Tissues, Bone” OR “Bony Apophyses” OR “Apophyses, Bony” OR “Bony Apophysis” OR “Apophysis, Bony” OR “Condyle” OR “Condyles” OR “Bones” OR “Bone”) AND (“Glomus Tumor” OR “Glomus Tumors” OR “Tumor, Glomus” OR “Tumors, Glomus” OR “Glomangioma” OR “Glomangiomas”) through December 2020. The inclusion criteria were as follows: case report(s) and case series, published in professional journals, and accessible full text. All the included paper was reselected independently by two of the authors reading the full text to meet the criteria: chief complaints were pain in or around the knee, finally diagnosed with glomus tumor.

### Data extraction and quality evaluation

Data extraction was independently performed and confirmed by two of the authors. The following information was extracted from included paper: authors, sex, affected side, family history and trauma history, locations, size of GTs, age at onset, illness duration, pathological types, treatments, follow-up, recurrence, and metastasis.

### Statistical analysis

The Mann–Whitney U test was used to determine the significance of age and duration of illness at diagnosis between males and females. Categorical data were presented as percentages and were compared by the Chi-squared test. The quoted P-values were two-sided, and a P-value < 0.05 was considered statistically significant. All statistical analyses were performed using SPSS 20.0 (SPSS Inc., Chicago, IL, USA). Data were processed with GraphPad (San Diego, California, USA) and 3Dbody (Shanghai QiaoMei Information Technology Co., Ltd, Shanghai, China).

### Literature review

**Fig. 2:**
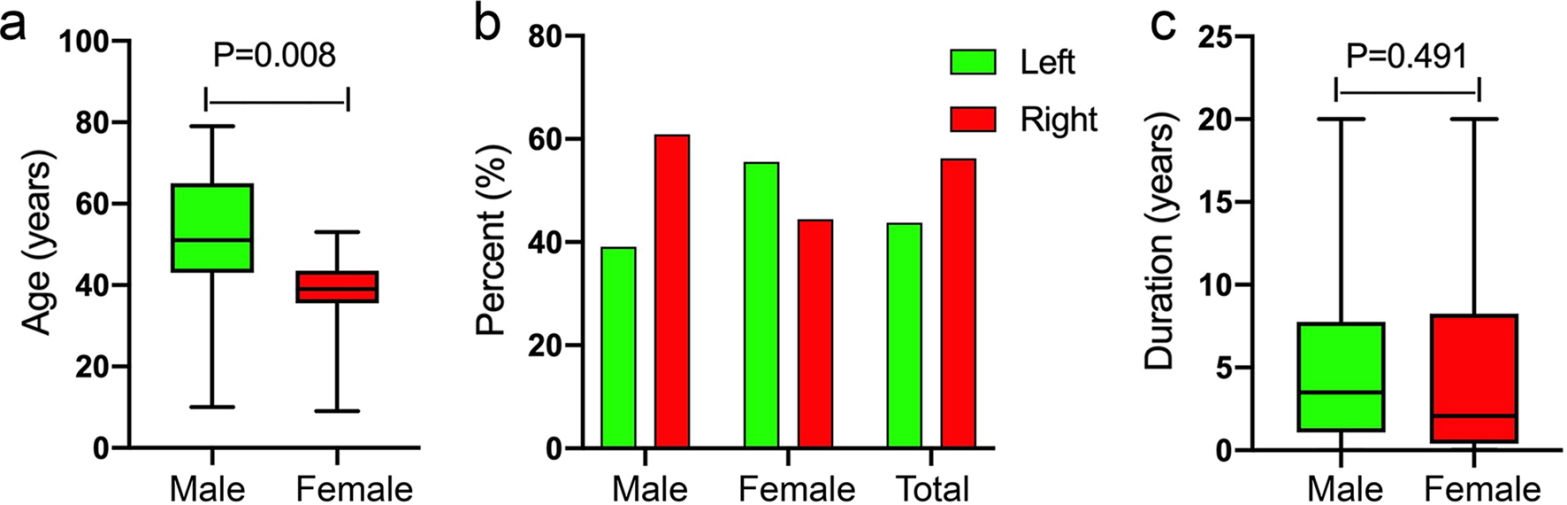
The age and duration of illness at diagnosis are presented as medians (interquartile ranges). **a** A significant age difference was found between male and female, P = 0.008. **b** The left and right knee incidence rate in male and female was found no statistical difference. **c** No significant difference in illness duration was found between male and female, P = 0.491

Around the knee GTs in males occurred significantly more frequently than in females (Table 1), although no significant difference was found in the prevalence of overall body GTs between men and women. Moreover, males with GTs were typically older than females (Fig. 2a). The median ages at definitive diagnosis were 51 and 39 for males and females, respectively. The upper and lower quartiles for males and females were 65 and 43, and 43.5 and 35.5, respectively. However, no statistically significant difference between males and females was found between affected left or right sides (Fig. 2b), or duration of illness (Fig. 2c). The illness duration ranged from 1 week to 20 years, and the median duration was 3.5 years for males, and 2.1 years for females. Close to 20% of all reported cases had a history of trauma, such as twisting the knee, automobile accident, a fall on the knee, and even total knee arthroplasty (Table 1). However, the specific causes for individual GTs are still unclear. The GT locations around the knee include the knee joint cavity, soft tissue around the knee (e.g. popliteal fossa, patellar tendon, iliotibial band, and Hoffa’s fat pad), and the distal femur and proximal tibia. The mean value of the largest diameter GTs was 21.6 mm, ranging from 4 to 65 mm. Additionally, the GTs reported with a size between 8 and 30 mm (the upper quartile and lower quartile), included the GT size in our case study. With the exception of one uncertain potentially malignant, and one definitely malignant GT, all other reported GTs were benign. Also, all the reported GTs were completely removed surgically, and only one recurred four times in the past 20 years [11].

**Table 1:** Previously reported glomus tumors in the knee area: side, cases, sex, history of trauma, location, size, age, duration of illness, and pathological category

Side	Cases	Sex	History	Location	Size (mm)	Age	Duration	Category
R [12]	1	F	None	Distal third of right vastus lateralis	15 × 30	31–40	16–20 yrs	Benign
L [13]	1	M	None	Inferior of medial tibial plateau	35 × 22 × 34	71–80	1–5 yrs	Benign
R [14] R [14]	2	M; F	Injured patella; Twist knee	Medial quadrant of the patella; Tibial tuberosity	10 × 10; 15 × 20	61–70; 41–50	1–5 yrs; 1–5 yrs	Benign; Benign
NR [7]	1	M	None	Subcutaneous tissue before the patella	60 × 50 × 50	71–80	several yrs	Uncertain malignant potential
NR [15]	1	NR	NR	Subcutaneous tissue in the medial aspect of the knee	20 × 10 × 20	51–60	16–20 yrs	Benign
L [16]	1	M	NR	Subcutaneous tissue before the patellar tendon	15 × 11 × 20	71–80	26–30 yrs	Benign
R [17]	1	F	NR	Hoffa’s fat pad	5 × 8	41–50	1–5 yrs	Benign
R [18]	1	M	None	Infrapatellar fat pad	10	61–70	1–5 wks	Benign
R [19]	1	M	None	Subcutaneous tissue of medial condyle of femur	50	71–80	1–5 yrs	Benign
L [20]	1	M	None	Subcutaneous tissue of lateral condyle of femur	7 × 6 × 4	51–60	1–5 yrs	Benign
R [21]	1	M	A fall on leg	Subcutaneous tissue of medial side of the knee	65 × 35 × 15	0–10	1–5 wks	Benign
L [22]	1	M	None	Subcutaneous tissue of the lateral collateral ligament	NR	51–60	6–10 yrs	Benign
R [23] R [23] R [23] NR [23]	4	M;M;M;M	None; None; NR; None	Medial to the patella tendon; Subcutaneous tissue superior and anterior to the tip of the fibula head; In the infrapatellar bursa; Subcutaneous tissue of the superolateral aspect of the patella	8 × 5; 15 × 15 × 12; 4–5 × 8 × 4; 20 × 8 × 4	41–50; 61–70; 61–70; 61–70	1–5 yrs; 6–10 mos; 1–5 yrs; NR	Benign; Benign; Benign; Benign
L [5]	1	M	NR	Lateral aspect of the intermediate third of the leg	24 × 20 × 20	41–50	6–10 yrs	Malignant
L [24]	1	M	Total knee replacement	Subcutaneous tissue of the anterolateral aspect of the knee	10 × 8 × 8	71–80	1–5 yrs	Benign
L [25]	1	M	Minor penetrating injuries	Subcutaneous tissue before the patella	55 × 43 × 27	41–50	1–5 yrs	Benign
L [26]	1	M	NR	Popliteal fossa	5 × 5	11–20	1–5 yrs	Benign
R [27]	1	M	NR	Anterior to the proximal pole of the patella	41 × 40	51–60	11–15 yrs	Benign
R [28] L [28] R [28]	3	M; F; M	None; None; None	Subcutaneous tissue before the patella; Subcutaneous tissue before the patellar tendon; Subcutaneous tissue before the quadriceps tendon	7 × 3; 4 × 4; 18 × 10	31–40; 41–50; 21–30	1–5 yrs; 1–5 yrs; 1–5 yrs	Benign; Benign; Benign
R [29]	1	M	None	In the patellar ligament	NR	41–50	6–10 yrs	Benign
R [30]	1	M	NR	Subcutaneous tissue of the lower third of the thigh	4	51–60	1–5 yrs	Benign
L [31]	1	F	None	Popliteal fossa	15 × 8	31–40	Several yrs	Benign
L [32]	1	F	Traffic accident	Confluent with the periosteum of the distal one-third of the posterior aspect of left femur	9 × 20	31–40	6–10 mos	Benign
L [33]	1	M	None	The periosteum and cortex of the lateral aspect of the femoral metaphysis below the iliotibial band	15 × 8	51–60	1–5 yrs	Benign
L [34]	1	M	NR	The surface of left posterior aspect of the distal tibia	30 × 20	51–60	6–10 yrs	Benign
R [11]	1	M	None	6 GTs in the gastrocnemius fascia	5 × (4—40)	51–60	16–20 yrs	Benign
R [35]	1	F	Twist ankle	In the midshaft of the fibula	40 × 25 × 15	31–40	1–5 mos	Benign
R [36]	1	M	None	In the plica synovialis of the lateral condylar side of knee	6 × 12 × 16	31–40	6–10 yrs	Benign
L [37]	1	F	None	Subcutaneous tissue of the deep posterior knee capsule	10 × 15 × 20	0–10	1–5 wks	Benign
R [38]	1	NR	None	Subcutaneous tissue behind Biceps femoris	NR	41–50	1–5 yrs	Benign
R	1	F	None	Confluent with the periosteum of femoral lateral supracondylar	10 × 7 × 4	41–50	6–10 yrs	Benign

*NA* not available, *NR* not record, *M* male, *F* female, *R* right, *L* left, *yr(s)* year(s), *wk(s)* week(s), *mo(s)* month(s)

The first record of a GT appears to be the description and management of eight painful subcutaneous tubercles by Wood in 1812 [39]. Masson, in an article published in 1924, is credited with first using the term GT to describe a tumor originating from a contractile neuromyoarterial body, also known as a glomus body [40]. GTs are reported to account for 1.6% of all soft tissue tumors in the extremities [41], and are most frequently found in the skin associated with the extremities. Approximately 65% of GTs are located in the subungual position, and are most commonly diagnosed between 20 and 40 years of age [42]. Around the knee GTs are definitively diagnosed between 38 and 59.5 years of age (the upper and lower age quartiles, Table 1). Classic subungual GTs occur more frequently in women, whereas ectopic locations are more common in men [10, 23], which is consistent with around the knee GTs. The first GT associated with the knee was described by Mabit in 1995 [29]. Unfortunately, most cases had experienced a long history of pain and/or knee joint limitations before obtaining a definitive diagnosis and subsequent surgical excision. The classic triad signs for GTs around the knee joint are not typical. Besides, the low incidence and unfamiliarity of orthopedic surgeons with GTs result in high rates of incorrect or missed diagnoses, and consequently delay correct treatment for patients with GTs.

### History of trauma

Based on our review, only 20% of all the reported GT cases had a history of trauma, including twisting the knee, automobile accident knee damage, a fall on the knee, or total knee replacement, and most of these traumas were easily diagnosed with meniscus injury, soft tissue hematoma, etc. The incidence of trauma in our study was significantly lower than that we found in a review of 8 cases, which reported a 50% incidence [25]. Notably, a GT caused knee pain 2 years after a successful total knee replacement due to osteoarthritis. Subsequently, a mass was detected in the subcutaneous tissue on the anterolateral aspect of the knee during an ultrasound examination. Immunohistochemistry and hematoxylin–eosin staining confirmed that the mass was a GT, even after complete surgical excision [24].

### Duration of illness

In our study, the illness duration upper and lower quartiles were 6.5 and 1 year, respectively. However, published reports describe the average illness duration between 7 and 11 years [43], which is significantly longer than our results. Because illness duration data are skewed, we used quartiles, instead of the mean, to describe the distribution. Although GTs are primarily individual tumors, there is a tendency for multiplicity in 10% of presentations [25], and the development of multiple GTs is associated with an autosomal-dominant mutation in the glomulin gene, located on chromosome 1p21-22 [14]. The incidence of multiple around the knee GTs is 2.8% (Table 1). In addition to common symptoms, some rare clinical manifestations were reported as follows. A 59-year-old man was diagnosed with tumor-induced osteomalacia, and a zone of activity in the left ankle area was found in an octreotide scan. An MRI scan revealed an in-homogenous lesion in the distal area of the left leg, posterior to the tibia. According to histopathological findings, the tumor was diagnosed as a GT [34]. Frumuseanu et al. reported a tumor in the medial aspect of the right knee, on a 10-year-old boy’s leg after a fall. The tumor developed outside the skin and became mushroom-like, but painless. Histopathological results finally confirmed a diagnosis of GT, with no local recurrence after complete tumor excision [21].

### Clinical manifestations

GTs typically present with the classic triad of pain and tenderness to palpation and hypersensitivity to cold. Many studies have found that extradigital GTs often present with pain but not temperature sensitivity, making these ambiguous lesions even harder to identify [27]. Most patients claim limited knee movement, accordingly, a diagnosis of knee osteoarthritis and soft tissue inflammation based on pain and limited movement is likely. Additionally, orthopedic surgeons should consider GTs for patients with chronic pain around the knee that is not relieved by non-steroidal anti-inflammatory and glucocorticoid drugs.

### Imaging results

Most patients initially underwent an x-ray examination with plain film. Although some GTs, especially confluent with the periosteum and eroding the cortical bone presented an abnormal signal, most X-ray results in other patients were negative. Since MRI is not a routine test for knee pain, it may explain why around the knee GTs are often misdiagnosed or missed altogether.

The preferred methods for diagnosing GTs are MRI and ultrasound [27]. An ultrasound may be the initial test of choice because of its low cost, speed, and convenience for the patient. The ultrasound GT results typically show GTs as round or ovoid hypoechoic masses, and they have been reported as hyper-vascular. MRI has proved to be the most sensitive imaging modality for GT diagnosis. GTs typically present with decreased signal intensity on T1-weighted images, and increased signal intensity on T2-weighted images. When clinical diagnosis suggests GT, a negative MRI result should not impede excision, because small size may be responsible for missed detection and false negative results, and MRI specificity has been estimated at approximately 50% [43].

**Fig. 3:**
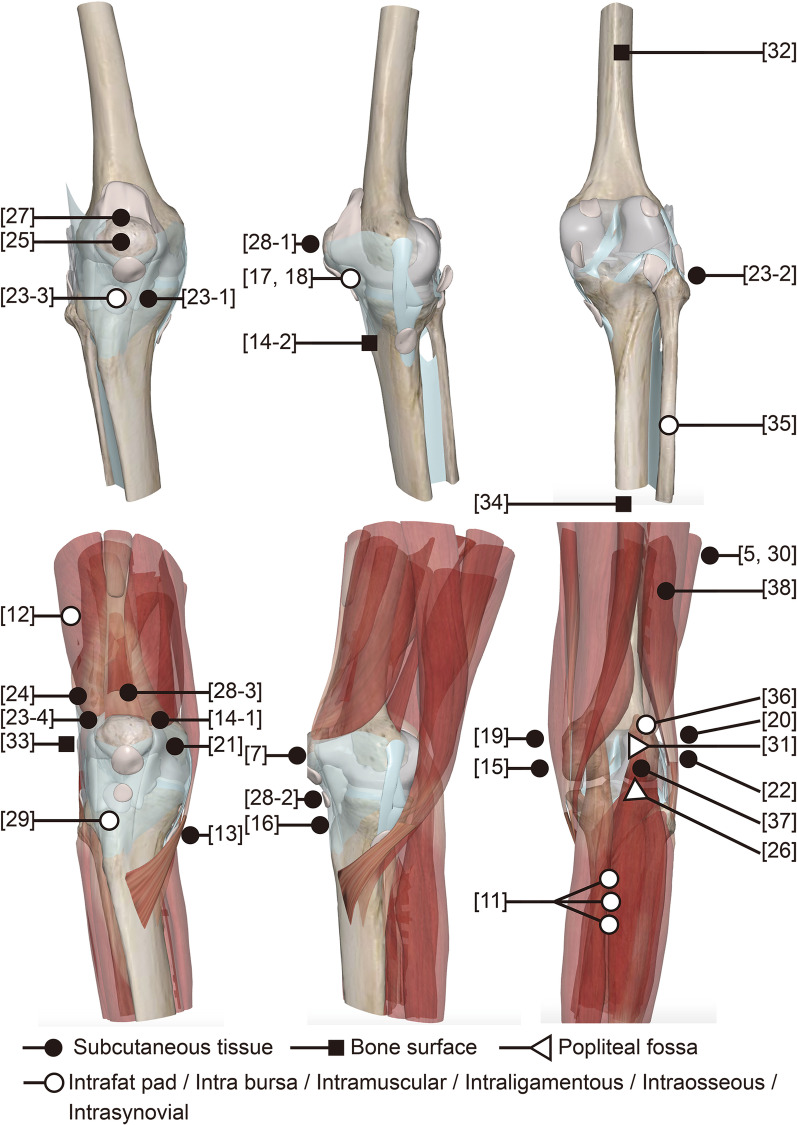
The most common locations for GTs around the knee. The structures of the knee were created by software 3Dbody anatomy. Arabic numerals represent the reference number reporting glomus tumors

The most common locations for GTs are subcutaneous tissue, muscle, synovium, fat pad, periosteum, and ligament (Fig. 3). However, while MRI may identify many GT characteristics, the differentiation of benign from malignant lesions, or uncertain potential malignancy is not absolute [42]. Imaging results should always be coupled with the size, location, borders, adjacent tissue involvement, and internal characteristics of the lesion to inform a differential diagnosis, and surgical planning [44]. The MRI results combined with clinical symptoms alone could not confirm a definitive diagnosis of a benign GT in our case study.

### Diagnosis and differential diagnosis

GTs are usually benign masses, and a 1% likelihood of malignancy does exist [25]. However, the incidence of uncertain potential malignancy and malignant around the knee GTs in our review were both 2.8%. Ample evidence is required to confirm the incidence of malignant GTs. Often, it is difficult to make a definitive diagnosis based only on clinical symptoms, signs, and MRI and ultrasound results. In our study, the definitive diagnosis was made after results were obtained from H&E staining coupled to immunohistochemical staining.

The differential diagnosis for knee pain is extensive. Only 9%–20% of extradigital GTs were correctly diagnosed by the primary physician [10]. The most common misdiagnosis were neurogenic tumors, vascular tumors, pigmented nevi, epidermal cysts, lipomas, leiomyomas, and sarcoidosis [10]. Moreover, GTs must be differentiated from Baker’s cyst, especially for knee pain in children. Small GTs beneath the patella must also be distinguished from sub-patellar fat pad inflammation based on MRI and/or ultrasound images.

### Therapy and prognosis

The typical GT presentation is a red–purple cutaneous nodule, usually causing pain out of proportion to size. Currently, the most effective therapy accepted for GTs is complete surgical excision. The reported recurrence rate after excision is approximately 10% [16], which is similar to the 7.1% in our review. After surgical excision, pain is usually relieved immediately, and knee range of motion is recovered. Inadequate excision may result in tumor recurrence within days to weeks, and symptoms may only appear 2–3 years postoperatively [45]. Radiotherapy may be a potential treatment for GTs in addition to surgical excision. Liu et al. reported a GT outside the T2 paravertebral space, and treatment with radiotherapy over 3 months was chosen due to financial considerations, but the GT recurred 1.5 years after treatment [46]. Consequently, further evidence is required to evaluate better treatments for GT.

More attention should be given to GT cases, particularly since they cause tremendous and long-term patient pain. In our study, the GT diagnosis was suggested by clinical manifestation and MRI and ultrasound results, and finally confirmed by histopathology results. Results from a review of the literature on GTs revealed that male patients were older than females, with a statistically significant difference. However, the affected sides, and duration of illness between male and female were not significantly different. A GT should be suspected after a trauma, but additional cases are required to determine the frequency of around the knee GT occurrence. Complete surgical excision is the most widely applied treatment, and recurrence rate is very low, since most GTs around the knee are benign. However, close attention should be given to differentiate uncertain potentially malignant, and malignant GTs from benign GTs. In conclusion, further evidence is required to elucidate the main causes and best treatments for GT.

## Data Availability

The data may be available from the corresponding author upon reasonable request.

## Abbreviations

GT(s): Glomus tumor(s)
NF1: Neurofibromatosis type 1
MRI: Magnetic resonance imaging
H&E: Hematoxylin–eosin
NA: Not available
NR: Not record
M: Male
F: Female
R: Right
L: Left
yr(s): Year(s)
wk(s): Week(s)
mo(s): Month(s)
